# Quadratus lumborum block (transmuscular approach) versus transversus abdominis plane block (unilateral subcostal approach) for perioperative analgesia in patients undergoing open nephrectomy: a randomized, double-blinded, controlled trial^☆^

**DOI:** 10.1016/j.bjane.2021.01.009

**Published:** 2021-03-21

**Authors:** Amany H. Saleh, Mai W. Abdallah, Ashraf M. Mahrous, Norhan A. Ali

**Affiliations:** Cairo University, Kasr Al Aini Hospital, Cairo, Egypt

**Keywords:** Open nephrectomy, Regional anesthesia, Visual Analogue Scale, Postoperative analgesia

## Abstract

**Study objective:**

Patients undergoing open nephrectomy surgery experience severe perioperative pain, which is primarily due to incision of several muscles. Abdominal wall blocks are known to reduce pain without causing epidural-associated hypotension. We conducted this study to compare unilateral ultrasound-guided transmuscular quadratus lumborum block and posterior transversus abdominis block in combination with general anesthesia alone in terms of intraoperative and postoperative analgesics and hemodynamics and postoperative complications.

**Methods:**

This was a randomized, double-blinded, controlled trial conducted in the operating room. This study included 48 patients aged 20–60 years, with ASA I and II, and a body mass index ≤ 30 kg.m^-2^ who were scheduled for open nephrectomy procedure.The 48 patients scheduled for nephrectomy were randomly allocated into one of the following three groups after induction of general anesthesia: Group A (n = 16) received USG transmuscular QLB; Group B (n = 16) received unilateral USG posterior transversus abdominis plane (TAP) block; and Group C (n = 16; control group) did not receive any blocks. Introperative fentanyl consumption, and hemodynamics (heart rate and mean arterial pressure (MAP)) were recorded after anesthesia induction, at surgical incision, and every 15 min till the end of surgery. Visual Analogue Scale (VAS) was evaluated immediately at 30 min and 1,2,4,6, and 12 hours postoperatively. The time of first analgesic request was also recorded.

**Results:**

Intraoperative fentanyl consumption (μg) was significantly lower in Groups A and B (164.69 ± 27.35 and 190.31 ± 44.48, respectively) than in Group C (347.50 ± 63.64) (*p* < 0.001). Postoperatively, total pethidine consumption was significantly lower in Groups A and B than in Group C (85.31 ± 6.68, 84.06 ± 4.17 mg, and 152.19 ± 43.43 mg, respectively) (*p* < 0.001. Time to rescue analgesia was longer in Groups A and B than in Group C (138.75 ± 52.39 min, 202.50 ± 72.25 min, and 37.50 ± 13.42 min, respectively) (*p* < 0.001). VAS score was significantly lower in Groups A and B than in Group C at 30 min and 1, 2, 4, and 6 hours postoperatively.

**Conclusion:**

Transmuscular quadratus lumborum block and posterior transversus abdominis blocks were effective in providing perioperative analgesia in patients undergoing open nephrectomy. However, quadratus lumborum block provided superior analgesia.

## Introduction

Severe perioperative pain experienced during and after surgical procedures performed by flank incision is primarily due to the incision of several muscles. Postoperative pain affects patients’ comfort and satisfaction and prolongs the duration of hospital stay and increases post-procedural complications.^1^ Although abdominal wall blocks are known to decrease opioid requirements without causing epidural-associated hypotension, their role in flank surgeries has been less well-established. The dermatomes that need to be covered in flank incision are T9 to T11.^2^

Studies have confirmed that ultrasound-guided (USG) transversus abdominis plane (TAP) block is an effective method of analgesia for upper abdominal surgeries,^3^ lower abdominal surgeries,^4^ and kidney transplantation,^5^ with minimal side effects.

A novel USG QL block is the transmuscular approach that depends on clearly identifiable sonographic bony landmarks. It can provide safe, easy, and effective analgesia covering from thoracic 7 to thoracic 12, lumbar 1. It is considered that the transmuscular QL block is effective for abdominal surgeries such as laparoscopy, inguinal hernia surgery, and femoral–femoral bypass.^6^

This study was designed to compare the success rate of unilateral ultrasound-guided transmuscular quadratus lumborum block with that of unilateral posterior ultrasound-guided TAP block in providing perioperative analgesia in patients undergoing open nephrectomy.

## Methods

**Figure 1:**
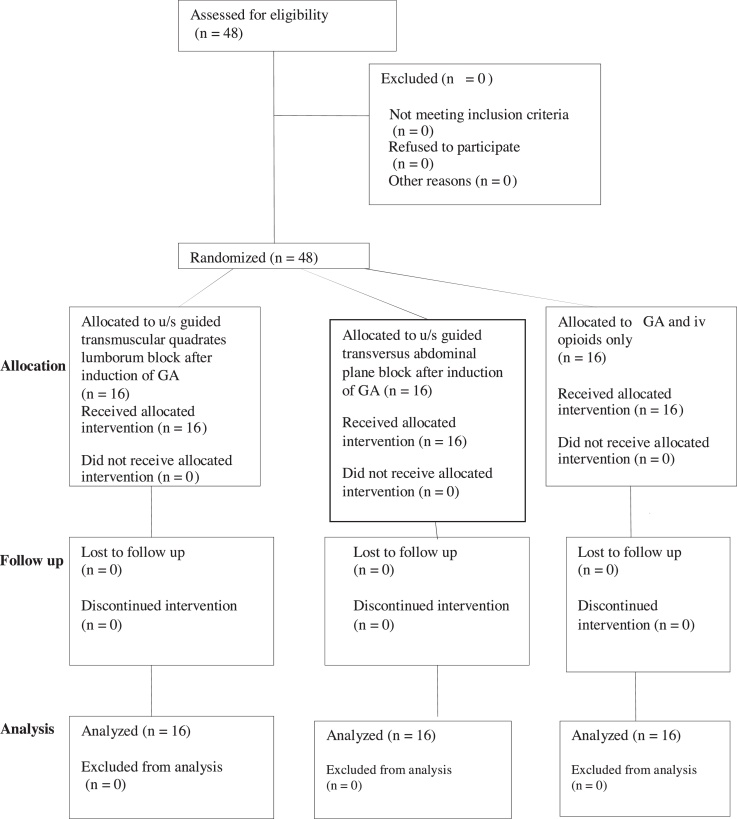
CONSORT chart.

This study was approved by the ethical committee of Cairo University (N-36-2018), and written informed consent was obtained from all patients. The study was registered as a randomized, double-blinded clinical trial at clinicaltrials.gov (NCT03744923, principal investigator: Amany Hassan, date of registration: November 2018) before patient enrollment. This manuscript adhered to the applicable CONSORT guidelines (Fig. 1). A total of 48 patients aged 20–60 years scheduled for open nephrectomy procedure, with ASA (American Society of Anesthesiologists) physical status I and II, and a body mass index ≤ 30 kg.m^-2^, were included. Patients refusing the use of regional blocks and having ASA III–VI, bleeding disorders, skin lesions or infection at the site of proposed needle insertion, allergy to amide local anesthetics, neurological disorders, and BMI > 30 kg.m^-2^ were excluded from the study.

Patients were allocated to the study groups using a computer-generated random list, and the group assignments were sealed in sequentially numbered opaque envelopes that were opened after the induction of anesthesia. The data collector was blinded to the group assignment and to the monitoring anesthetist, and the person who performed the block was not the data collector (double-blinded).

The anesthesiologist met the guardians and informed consent was obtained, after which the patients were examined, followed by all routine investigations. On arrival to the operating room, an intravenous (IV) cannula 20G was inserted, and Ringer's acetate solution was infused. A five-lead electrocardiogram, a pulse oximeter, and a noninvasive blood pressure cuff were applied. Then, midazolam 0.025 mg.kg^-1^ was administered.

Patients were randomly allocated into one of the following three groups: Group A (n = 16): patients received USG transmuscular quadratus lumborum block (QLB) after induction of general anesthesia; Group B (n = 16): patients received unilateral USG posterior TAP block after induction of general anesthesia. Group C (n = 16) (control group) did not receive any blocks.

Next, general anesthesia was induced using propofol 2 mg.kg^-1^ over 20–30 s, fentanyl 2 μg.kg^-1^, and atracurium 0.5 mg.kg^-1^ to facilitate endotracheal intubation. Anesthesia was maintained using isoflurane 1.5% and atracurium infusion at a dose of 0.3 mg.kg^-1^.h^-1^. For all patients, strict fluid management was followed according to body weight, and blood loss was adequately estimated and replaced.

### Group A (transmuscular QLB)

The patient was positioned in the lateral position, with the side to be anesthetized being located upward. Complete aseptic precautions were taken by wearing sterile gown and sterile gloves and sterilizing the site of the block.

An ultrasound device was used, in which a convex probe with broadband (5–8 MHz) covered with sterile plastic sheath was placed in the mid-axillary line cranially to the iliac crest to identify the three muscles of the anterior abdominal wall (transversus abdominis, internal oblique, and external oblique). Then, scan dorsally to keep the transverse orientation until seeing the aponeurosis of the transverses abdominus muscle and by following this aponeurosis, QL muscle was visualized with its attachment to the lateral edge of the transverse process of the L2 vertebral body and visualize the thoracolumbar fascia at the lateral edge of the QL muscle. The view of the psoas major muscle anteriorly, the erector spinae muscle posteriorly, and the QL muscle adherent to the apex of the transverse process result in a well-known view of a Shamrock with three leaves (trifoliate).

A spinal needle (20G) was inserted in-plane from the posterior to anterior direction, and the tip of the needle was advanced through the QL muscle, penetrating the ventral proper fascia of the QL muscle. The target site for injection was the plane between the quadratus lumborum and psoas major muscles. A test dose of 1 mL of 2% lidocaine was injected for hydrovisualization of the needle-tip position and for confirming its correct positioning. This was followed by injection of 0.5% bupivacaine (0.25 mL.kg^-1^) and 2% lidocaine (0.15 mL.kg^-1^) together. The dose of both agents did not exceed the safe limits (2 mg.kg^-1^ for bupivacaine and 3 mg.kg^-1^ for lidocaine).

### Group B (TAP block group)

The patient was positioned in the supine position. The probe was positioned transversely midway between the iliac crest and the costal margin at the level of the mid-axillary line. Once the external oblique muscle (EOM), internal oblique muscle (IOM), and transversus abdominis muscle (TAM) were visualized at the level of the posterior axillary line between the 12^th^ rib and the iliac crest with visualization of the anterior pole of the QL muscle, the puncture area and the ultrasound probe were prepared in a sterile manner. A 20G spinal needle was directed to approach the TAP using the “in-plane” USG-guided technique. Once the tip of the needle was placed in the space between the IOM and TAM at the closest point to the QL muscle, a 1 mL test dose of 2% lidocaine was injected for hydrovisualization of the needle-tip position and for confirming its correct positioning. This was followed by injection of 0.5% bupivacaine (0.25 mL.kg^-1^) and 2% lidocaine (0.15 mL.kg^-1^) together. The dose of both agents did not exceed the safe limits (2 mg.kg^-1^ for bupivacaine and 3 mg.kg^-1^ for lidocaine). The drug was observed as a dark oval shape spreading in the TAP.

In the three groups, skin incision was delayed for 10 minutes after performing the block to ensure its spread and efficacy. The observing anesthetist was advised to inject fentanyl increment doses (0.5 μg.kg^-1^) when the mean blood pressure, heart rate, or both increased by > 20% from the baseline.

At the end of surgery, inhalational anesthetics were discontinued, the muscle relaxant was reversed with atropine (0.01 mg.kg^-1^) and neostigmine (0.05 mg.kg^-1^), spontaneous breathing was allowed, the endotracheal tube (ETT) was removed, and then the patient was transferred to postanesthesia care unit.

Postoperatively, all patients received IV paracetamol 1 g every 8 hours. As a rescue analgesic, meperidine (0.5 mg.kg^-1^) was used when the Visual Analogue Scale (VAS) was > 4.

### Primary outcome

The primary outcome of our study was measurement of the total amount of fentanyl consumption during the intraoperative period in the three allocated groups as an indicator of hemodynamic stability and efficacy of both types of blocks.

### Secondary outcomes

The secondary outcomes were as follows: hemodynamic stability (mean blood pressure and heart rate not exceeding 20% of baseline levels), meperidine consumption over 12 hours in all groups, estimation of the time to rescue analgesia in all groups, patient satisfaction (measured using pain scores at rest and during movement according to the VAS score; a VAS score < 4 will be considered as satisfactory), ease of performing the respective blocks (assessed using the time required to perform the blocks in minutes and number of attempts required to reach the plane of block), comparison of both types of blocks in providing adequate analgesia perioperatively (assessed using fentanyl consumption intraoperatively and meperidine consumption and VAS score postoperatively in the groups of both blocks). Postoperative complications were also recorded in both groups (bowel injury, injury of the kidney, hematoma formation, intravascular injection, pruritus, nausea, vomiting).

### Statistical analysis

Data were coded and entered using the statistical package SPSS version 25. Data were summarized using mean and standard deviation or median and interquartile range for quantitative variables and frequencies (number of cases), and relative frequencies (percentages) for categorical variables. Between-group comparisons were conducted using unpaired *t*-test or analysis of variance with multiple comparison post hoc test for normally distributed quantitative variables, whereas nonparametric Kruskal–Wallis test and Mann–Whitney test were used for non-normally distributed quantitative variables.^7^ Chi-square (χ^2^) test was performed for assessing categorical data. Exact test was used instead when the expected frequency is less than 5.^8^ The Kaplan–Meier analysis was performed to assess the duration of analgesia. Significance values were adjusted by the Benferroni correction for multiple tests. *P*-values < 0.05 were considered statistically significant.

### Sample size calculation

Our primary outcome was intraoperative fentanyl consumption. A previous study reported an intraoperative fentanyl consumption amount of 580 (91) μgin this operation.^3^ Sample size was calculated using the MedCalc software to detect a mean difference of 15% (58 μg) between study groups. A minimum number of 45 patients (15 patients per group) was required to reach a study power of 90% and an alpha error of 0.05. The number was increased to 48 patients (16 patients per group) to compensate for possible dropouts.

## Results

A total of 48 patients who were scheduled for open nephrectomy surgeries were enrolled in our study. These patients were randomly allocated into three equal groups using the closed envelope method. After the induction of general anesthesia, Group A received ultrasound-guided transmuscular QL block, Group B received ultrasound-guided posterior TAP block, and Group C received only general anesthesia.

**Figure 2:**
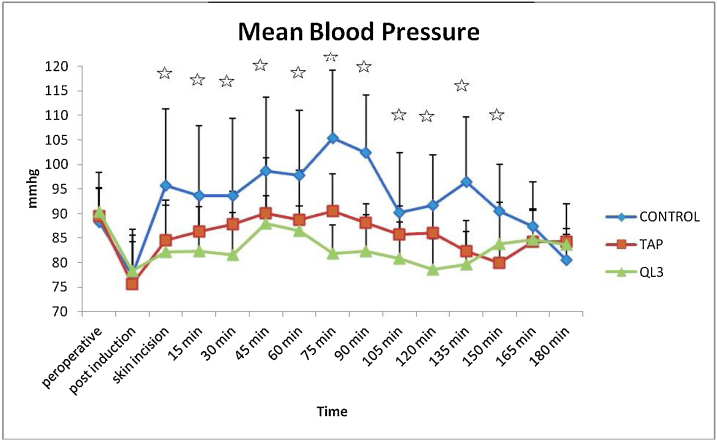
Comparison of intraoperative mean blood pressure between the three groups. TAP, transversus abdominis plane. *P*-value < 0.05 is considered significant.

**Figure 3:**
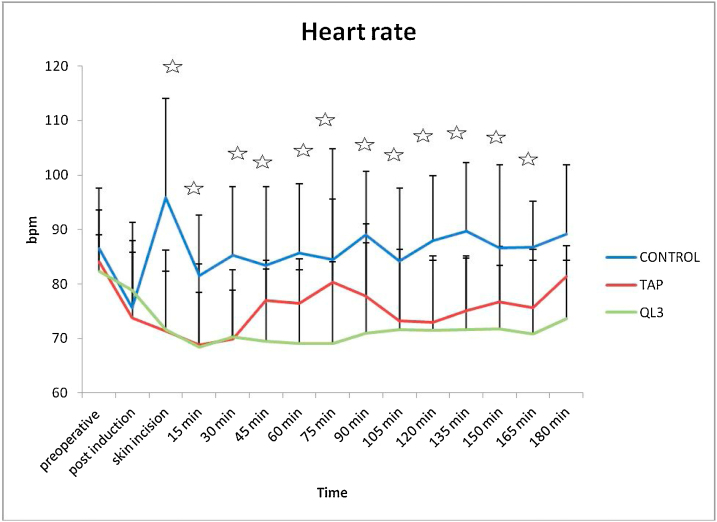
Comparison of intraoperative heart rate between the 3 groups. TAP, transversus abdominis plane. *P*-value < 0.05 is considered significant.

There were no statistically significant differences in the demographic data of the patients (Table 1). The time to perform TAP block was less than that required for quadratus lumborum block, with a statistically significant difference (Table 2). The mean arterial pressure (MAP) (Fig. 2) measured intraoperatively revealed significant differences between the three groups. Statistically significant differences were also observed in the intraoperative heart rate between the groups (Fig. 3). The average heart rate and MAP measurements over the 12-hours postoperative period exhibited significant differences between the three groups (Table 2).

**Table 1:** Patients characteristics and duration of operation.

		**Control group**		**TAP group**	**QL3 group**
		**Mean (SD)**	**Mean (SD)**	**Mean (SD)**	***p***-**value**
**Sex**	Female	5 (31.2%)	10 (62.5%)	6 (37.5%)	0.169
	Male	11 (68.8%)	6 (37.5%)	10 (62.5%)	
**Age**	40.31 (7.22)	40.19 (5.65)	42.69 (9.97)	0.598	
**Weight**	82.50 (14.26)	78.44 (13.87)	77.88(14.09)	0.601	
**Duration of operation (min)**	151.88 (26.20)	143.44 (25.67)	150.94 (29.22)	0.630	

TAP, transversus abdominis plane.

For sex, data was represented as count and percentage. For others, Data was expressed as mean and standard deviation. *P-*value < 0.05 is considered significant.

**Table 2:** Intraoperative fentanyl consumption and times of administration of fentanyl between, mean postoperative MAP and HR over 12 hours in and time to perform the block.

	**Control group**	**TAP group**	**QL3 group**	
	**Mean (SD)**	**Mean (SD)**	**Mean (SD)**	***p-*****value**
**Number of fentanyl times administration (induction dose excluded)**	4.56 (0.96)	0.87 (0.96)	0.25 (0.45)	< 0.001
**Total fentanyl consumption (mic)**	347.50 (63.64)	190.31 (44.48)	164.69 (27.35)	< 0.001
**Postoperative HR**	97.62 (8.41)	78.38 (8.06)	75.50 (8.37)	< 0.001
**Postoperative MABP**	97.00 (10.77)	85.38 (7.54)	87.69 (3.72)	< 0.001
**Time to perform block**(**min)**	-	3.56 (1.03)	4.44 (0.81)	0.012

TAP, transversus abdominis plane.

Data expressed as mean and standard deviation. *P*-value < 0.05 is considered significant.

The number of times of fentanyl administration and the amount of fentanyl consumption during the intraoperative period are shown in Table 2. Statistically significant between-group and within-group differences were observed in these parameters, with the lowest finding being observed in the QL3 group. The number of times of fentanyl administration and the total amount of fentanyl consumption were less in the QL3 group than in the TAP group, but there were no statistically significant differences (Table 3).

**Table 3:** Comparison between three groups with regarding nausea and vomiting.

		**Control group**		**TAP group**		**QL3 group**		***p***-**value**
		**Count**	%	**Count**	**%**	**Count**	%	
PONV	Yes	**12**	75.0%	**3**	**18.8%**	**2**	12.5%	< 0.001
	No	**4**	25.0%	**13**	**81.2%**	**14**	87.5%	

TAP, transversus abdominis plane.

Data is expressed in terms of count and percentage. *P*-value < 0.05 is considered significant.

**Figure 4:**
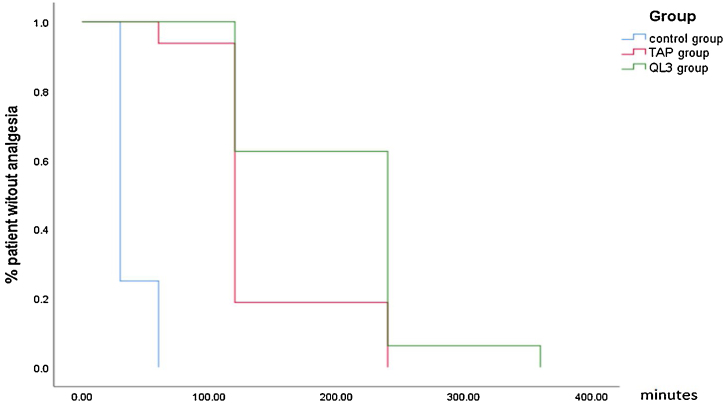
Kaplan–Meier curve representing comparison of time of rescue analgesia between the three groups. TAP, transversus abdominis plane.

The time to first request of rescue analgesia was statistically significantly longer in both QL3 and TAP groups than in the control group, as shown in the Kaplan–Meier graph (Fig. 4). The total amount of pethidine consumption and the number of times of pethidine administration were statistically significantly lower in the QL3 and TAP groups than in the control group.

Regarding the time to rescue analgesia, number of times of pethidine administration, and total amount of pethidine consumption between the QL3 and TAP groups, it was observed that the time to rescue analgesia was statistically significantly prolonged in the QL3 group. However, the number of times of pethidine administration and the total amount of pethidine consumption were not significant.

**Figure 5:**
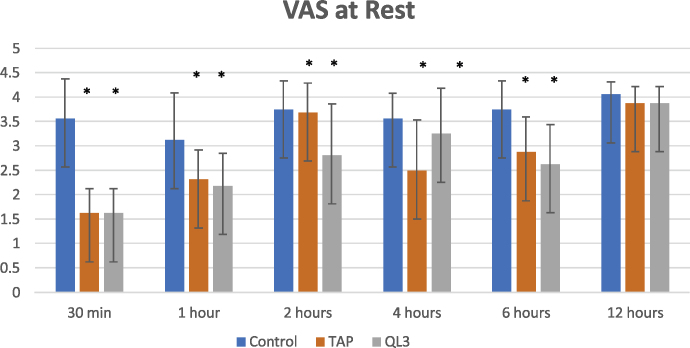
VAS score at rest between three groups at different study times. Data expressed as mean and Standard deviation. VAS, visual analogue scale; TAP, transversus abdominis plane.

**Figure 6:**
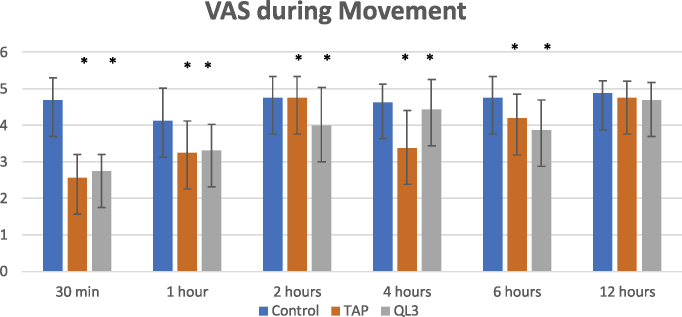
VAS scores during movement between the three groups at different study time points. Data are expressed as mean and standard deviation. VAS, visual analogue scale; TAP, transversus abdominis plane.

Regarding the VAS pain scores at rest and during movement, there were statistically significant differences between the three groups at all study time points, except at 12 hours postoperatively (Figure 5, Figure 6). VAS score was statistically significantly lower in the QL3 group than in the TAP group at 2 hours postoperatively. It was also statistically significantly lower in the TAP group than in the QL3 group at 4 hours postoperatively.

Postoperative nausea and vomiting (PONV) were observed in 12 patients in the control group, whereas only 3 and 2 patients in TAP and QL3 groups experienced PONV, respectively (*p* < 0.001). None of our study patients experienced any other complications such as bowel injury, injury of the kidney, hematoma formation, intravascular injection, and pruritus.

## Discussion

This study compared the efficacy of unilateral ultrasound-guided transmuscular quadratus lumborum block (Group A) and unilateral posterior ultrasound-guided TAP block (Group B) in combination with general anesthesia with that of general anesthesia alone (control; Group C) to evaluate the success rate of providing perioperative analgesia in patients undergoing open nephrectomy.

To our knowledge, this is the first study to compare the efficacy of quadratus lumborum block and TAP block in providing intraoperative and postoperative analgesia in patients undergoing open nephrectomy surgeries.

The results of this study were divided into intraoperative and postoperative findings. The time to perform the block was significantly lower in Group B (3.56 ± 1.03 min) than in Group A (4.44 ± 0.81 min). Intraoperatively, both groups (A and B) exhibited lower recordings in heart rate (HR) and MAP intraoperatively than the control group, with statistically significant differences. Moreover, both groups (A and B) showed high analgesic efficacy with significant reduction in fentanyl consumption (164.69 ± 27.35 and 190.31 ± 44.48 μg, respectively) compared with group C (347.50 ± 63.64 μg). Group A exhibited lower fentanyl consumption than Group B; however, this was not statistically significant. The number of times of fentanyl administration was also significantly lower in Groups A and B than in Group C.

Our results were consistent with those reported by Shafeek et al. who conducted a study comparing the analgesic efficacy of ultrasound-guided transmuscular quadratus lumborum block (QL3) with that of TAP block and general anesthesia with intravenous opioid drugs during laparoscopic bariatric surgery and in the early postoperative period. Their results revealed statistically significant differences between the two block groups and the control group in terms of intraoperative hemodynamic changes (mean blood pressure and heart rate). Moreover, the GA group had the highest pain scores, shorter time of first request of rescue analgesia, and more consumption of morphine than the groups treated with quadratus lumborum block and TAP block.^9^

In addition, Elsharkawy et al. investigated the subcostal approach to anterior quadratus lumborum block in providing analgesia in patients undergoing open urological surgeries. Their findings revealed that the subcostal approach to anterior QL block provided adequate analgesia between T6–7 and L1–2, which was required for analgesia following open urological surgical procedures. This is consistent with the findings of our study indicating the efficacy of transmuscular quadratus lumborum block in providing analgesia in patients undergoing flank incision surgeries.^10^

Our study findings are also consistent with those reported by Yousef NK who compared bilateral TAP block versus QLB in 60 patients undergoing total abdominal hysterectomy under GA. They demonstrated that the QLB group consumed less intraoperative fentanyl than the TAP group (43.16 ± 19.5 μg in QLB vs. 110.6 ± 22.4 μg in TAP). Despite showing significance that was not obviously evident in our study, their study result supported the final finding of our study that QL block is more effective than TAP block.^11^

Postoperatively, with respect to VAS score, our study demonstrated that the time to first request of rescue analgesia was the longest in Group A (202.50 ± 72.25 min) compared to that in Group B (138.75 ± 52.39 min) and Group C (37.50 ± 13.42 min). The total amount of pethidine consumption during the total 12 hours postoperatively was similar in Groups A and B (84.06 ± 14.17 and 85.31 ± 16.68 mg, respectively), which was significantly lower than that in Group C (152.19 ± 43.43 mg). The number of times of pethidine administration in the 12-h period showed the same relationship. The VAS score was significantly lower in Groups A and B over the 12-h period than that in Group C during rest and movement. VAS score was high in Group B compared with Group A at 2 hours, which necessitated rescue analgesia. At 4 hours, Group A showed a higher VAS score than group B, which could be attributed to the early administration of pethidine to Group B.

Regarding complications, more patients experienced PONV in Group C (12 patients) than in Group B (3 patients) and Group A (2 patients).

Our study findings are consistent with those of Yousef NK who reported shorter time of analgesia and more morphine consumption in the TAP group than in the QLB group.^11^

In the above-mentioned study of Shafeek et al., their results were consistent with our results regarding the time to first call for rescue analgesia (quadratus lumborum group 187.66 ± 23.84 vs. 202.50 ± 72.25 min in our study, and TAP group 128.07 ± 15.25 vs.138.75 ± 52.39 min in our study). Furthermore, VAS pain score and PONV comparison were significant in the early postoperative period until 6 hours postoperatively in the comparison between the three groups. Total morphine consumption amount was significantly less in the QL3 group than in the TAP group in their study, which contrasts with our study finding. However, they finally concluded that TAP block was more effective than intravenous opioid drug analgesia, and quadratus lumborum block was more effective than TAP block.^9^

Another double-blinded, randomized, controlled study whose findings were consistent with our study findings was performed by Blanco et al. in 50 patients undergoing elective cesarean section under spinal anesthesia. In this study, the first group received a QLB with bupivacaine, and the second group received a QLB with normal saline. Patients in the local anesthetic group showed lower consumption of morphine than the control group (*p* < 0.001) at 6 and 12 hours after the cesarean section. The VAS score was lower in the local anesthetic group than the control group at rest and during movement at all time points, except at 24 hours after the cesarean section.^12^

A case series study was performed by Warusawitharana et al. to investigate ultrasound-guided continuous transmuscular quadratus lumborum analgesia for open renal surgery (mean morphine consumption 50 mg over 48 hours) compared with wound infusion analgesia (93 mg morphine over 48 hours). Their study findings added further support to our study regarding the effectiveness of transmuscular quadratus lumborum block in flank incision procedures.^13^

In another study, Baidya et al. examined the effect of transmuscular quadratus lumborum block for perioperative analgesia in children undergoing pyeloplasty. They reported that transmuscular QL block provided satisfying postoperative analgesia in children undergoing lumbotomy and pyeloplasty surgeries.^14^

Our study findings are also consistent with those reported by Öksüz et al., who performed a randomized controlled trial to compare quadratus lumborum block and TAP block in 50 pediatric patients undergoing lower abdominal surgeries after general anesthesia induction. They reported a lower number of patients requiring analgesia in the first 24 hours postoperatively.^15^

In contrast to our study, a prospective, randomized, double-blinded study was reported by Kumar et al. in 70 adult patients undergoing lower abdominal surgeries. The patients were allocated into 2 groups –quadratus lumborum group and TAP group. The time for the first analgesic requirement was 243 ± 97.36 min in the TAP group and 447.00 ± 62.52 min in the quadratus lumborum group. The total amount of morphine consumption was 5.65 ± 1.55 mg in the TAP group and 3.25 ± 0.78 mg in the quadratus lumborum group, with the difference being statistically significant. This difference was explained in our study based on the effect of surgical incision, which opened the plane where the local anesthetic was injected, spilling a part of the injected volume and thus decreasing its effective time postoperatively. In addition, the use of morphine in that study as rescue analgesia compared to pethidine used in our study resulted in longer duration of action. Moreover, significant differences in postoperative pain scores at rest were observed between both groups up to 16 hours.^16^

### Limitations

The flank incision opened the plane where the local anesthetic was injected in both block groups, thus limiting its postoperative efficacy. Moreover, the period of testing of postoperative analgesia was limited to the first 12 hours postoperatively. However, both quadratus lumborum block and TAP block are known to produce analgesia for at least 48 hours postoperatively. In addition, the exact extent of the blocks was not clinically evaluated as the patients received the blocks after the induction of general anesthesia. The fixed intervals to evaluate VAS scores did not allow the maximum accuracy of determination of analgesia requirements of patients.

### Recommendations

It is recommended that further studies be performed on a larger number of patients to confirm our study findings. Longer postoperative follow-up period up to 48 hours is recommended for better assessment of the postoperative analgesic effects of both blocks. Moreover, introduction of a catheter with continuous infusion of local anesthetics appears to provide potentially strong postoperative analgesia. The catheter can be introduced percutaneously or through a surgical incision in situ.

## Conclusion

Transmuscular quadratus lumborum block provides more effective perioperative analgesia than posterior TAP block in patients undergoing nephrectomy through flank incision. However, both blocks are effective in achieving pain control perioperatively, with significant reduction of opioid consumption compared with the control group.

## Conflicts of interest

The authors declare no conflicts of interest.
